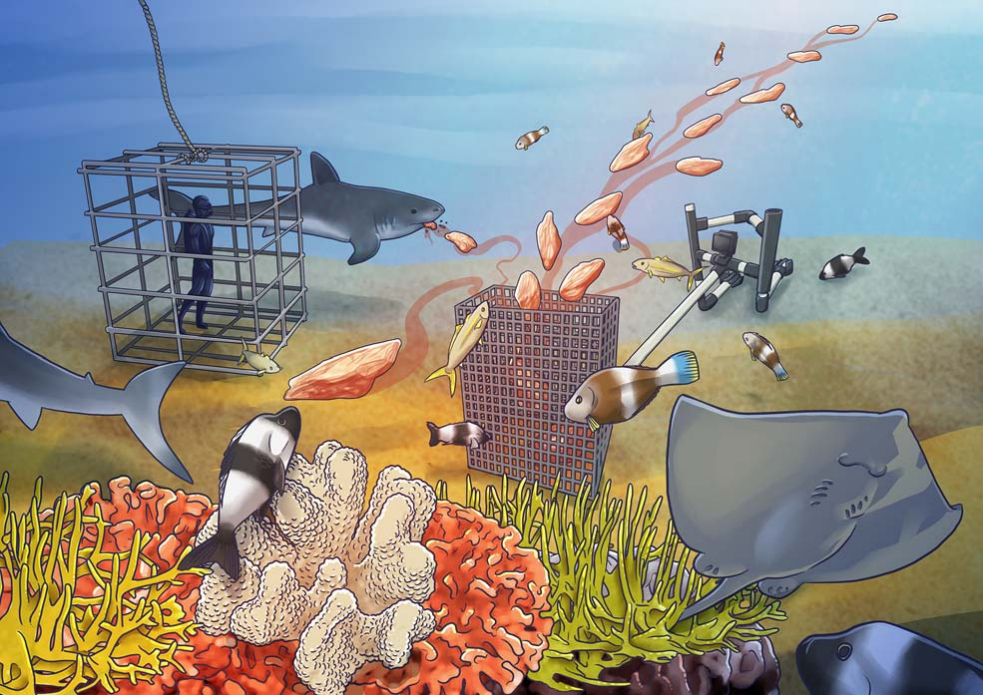# Cannot see the great white shark? Bait for it

**DOI:** 10.1093/conphys/coz114

**Published:** 2020-02-06

**Authors:** Björn Illing

**Affiliations:** ARC Centre of Excellence for Coral Reef Studies, James Cook University, Townsville, Queensland 4811, Australia

Cage diving to view great white sharks and other iconic marine mega-fauna is a huge part of the marine wildlife tourism industry. But cage diving is considered a double-edged sword. On one hand, it fosters positive conservation ethics in tourists and raises awareness. However, on the other hand, there are concerns about animal welfare, costs to shark fitness and disruption of the natural ecology of habitats, mostly related to the use of bait to attract sharks. In fact, at cage-diving sites that use bait, Lauren Meyer and her colleagues determined that the swimming behaviour and daily activity levels of great white sharks are affected but not their nutrition. However, Meyer *et al.* (2019) have just investigated the impact on the other fishes, including rays that are unintentionally fed at these sites, and it turns out, those fishes do experience the effects.

In their study, Meyer’s team (2019) chose two sites with different intensities of cage-diving activity and compared them with a control site without any cage diving. They tested whether non-targeted groups of fishes, including rays, ate the great white sharks’ bait (mostly fish remains) and if the fishes received a nutritional benefit from those provisions. To do this, the authors sampled several fish species from all sites and used biomarkers to assess if the bait was consumed. For example, they checked the gut contents to see if there was an immediate food intake, and they used fatty acid and stable isotope analyses to determine if the bait had been consumed at some point in history. The latter two methods use biochemical signatures in the food that are passed to the consumers. This way, the authors were able to track the provisions through time and up the food web. Simply put, the researchers could determine who ate what. The researchers also used remote underwater cameras with bait during times of no tourism operation, which helped the team assess how many fishes were present at each site. Meyer *et al.* (2019) found that pelagic fishes (e.g. silver trevally and yellowtail kingfish) nutritionally benefitted from the bait but this depended on how much bait was consumed. They also found that benthic fishes, such as several coral reef fishes and rays, even received some of the provisions. This highlights that the effects of bait feeding could even extend to species not directly observed to feed on the bait.

So where should the practice of using bait to attract marine mega-fauna, such as the great white shark, go in the future? This team of scientists highlights that ecosystem-wide impacts need to be assessed through comprehensive approaches, as there is a multitude of possible ecological consequences and not just to the sharks of interest. For example, the fish that unintentionally receive food should be monitored to see if provisioning changes their abundance or influences their natural diet in any way. Some pelagic fishes, such as silver trevally, feed heavily on the provisions and moderate the effects of using bait. So adjusting the amount of shark provisions to the abundances of non-target fish species could be a practicable compromise between managing popular cage-diving activities and ecosystem health. Furthermore, future research could also help identify the most resilient sites that are best suited for marine wildlife tourism.

Illustration by Erin Walsh; Email: ewalsh.sci@gmail.com